# Development and validation of a nomogram for predicting *Mycoplasma* *pneumoniae* pneumonia in adults

**DOI:** 10.1038/s41598-022-26565-5

**Published:** 2022-12-17

**Authors:** Yuan Ren, Yan Wang, Ruifeng Liang, Binwei Hao, Hongxia Wang, Jianwei Yuan, Lin Wang, Zhizun Guo, Jianwei Zhang

**Affiliations:** 1 Department of Environmental Health, School of Public Health, Shanxi Medical University, Taiyuan, 030001 Shanxi China; 2 Department of Infectious Disease, Shanxi Bethune Hospital, Shanxi Academy of Medical Sciences, Tongji Shanxi Hospital, Third Hospital of Shanxi Medical University, Taiyuan, 030032 Shanxi China; 3 Department of Pulmonary and Critical Care Medicine, Shanxi Bethune Hospital, Shanxi Academy of Medical Sciences, Tongji Shanxi Hospital, Third Hospital of Shanxi Medical University, Taiyuan, 030032 Shanxi China

**Keywords:** Biomarkers, Diseases, Health care, Medical research, Risk factors

## Abstract

The study aimed to explore predictors of *Mycoplasma pneumoniae pneumonia* (MPP) in adults and develop a nomogram predictive model in order to identify high-risk patients early. We retrospectively analysed the clinical data of a total of 337 adult patients with community-acquired pneumonia (CAP) and divided them into MPP and non-MPP groups according to whether they were infected with MP. Univariate and multivariate logistic regression analyses were used to screen independent predictors of MPP in adults and to developed a nomogram model. Receiver operating characteristic (ROC) curve, calibration curve, concordance index (C-index), and decision curve analysis (DCA) were used for the validation of the evaluation model. Finally, the nomogram was further evaluated by internal verification. Age, body temperature, dry cough, dizziness, CRP and tree-in-bud sign were independent predictors of MPP in adults *(P* < *0.05*). The nomogram showed high accuracy with C-index of 0.836 and well-fitted calibration curves in both the training and validation sets. The area under the receiver operating curve (AUROC) was 0.829 (95% CI 0.774–0.883) for the training set and 0.847 (95% CI 0.768–0.925) for the validation set. This nomogram prediction model can accurately predict the risk of MPP occurrence in adults, which helps clinicians identify high-risk patients at an early stage and make drug selection and clinical decisions.

## Introduction

Community-acquired pneumonia (CAP) is an infectious disease that has high morbidity and mortality rates^1^. CAP kills nearly one million adults each year in Asia^2^. *Mycoplasma pneumoniae* (MP) is the main pathogen causing CAP. *Mycoplasma pneumoniae* pneumonia (MPP) is a respiratory tract infection caused by MP that occurs mostly in children, accounting for approximately 10 to 40% of paediatric CAP cases^3,4^. In recent years, the incidence of MP infections within CAP has increased in adults. MPP may cause severe pulmonary complications, such as mechanized pneumonia, chronic interstitial fibrosis, and acute respiratory distress syndrome^5^. It may also affect multiple extrapulmonary systems, such as the central nervous system, cardiovascular system, digestive system, genitourinary system, and haematologic system and may even cause death^6–9^. An early diagnosis of MPP can improve the prognosis of the disease.

Currently, pathogen culture is the gold standard for the diagnosis of MP infection but requires at least 10–14 days; therefore, it does not allow for early and rapid diagnosis of the disease. Serological testing requires the collection of serum samples from acute and convalescent patients, takes 2–4 weeks and provides a retrospective diagnosis only^3^. PCR requires expensive equipment, the results are susceptible to the sample quality and collection technique, and false-positive results may occur^10,11^. Thus, a rapid, convenient and accurate tool for predicting MPP is needed.

Nomograms, which are intuitive and visualized risk prediction models, have been widely used in recent years to predict cancer and surgical prognosis^12–14^, can rapidly and accurately identify high-risk groups and can improve the diagnostic efficacy. In the latest research, a nomogram was used to predict the occurrence of refractory *Mycoplasma pneumoniae* pneumonia (RMPP) in children and bronchial mucus plugs in children with MPP, and both showed good predictive performance^15,16^. However, few predictive models have been reported for MPP in adults. In this study, we aimed to explore the predictors of MPP in adults and to develop a simple and practical nomogram prediction model to help clinicians accurately identify patients at high risk of MPP early and to assist them in medication selection and clinical decision-making.

## Materials and methods

### Study population

We retrospectively collected clinical data from all adult patients diagnosed with CAP at Shanxi Bethune Hospital from January 2021 to December 2021. A total of 337 patients were finally included in the study and were divided into MPP group and non-MPP group according to whether they were infected with MP. The inclusion criteria were as follows: (1) age ≥ 18 years old and (2) fulfilled the diagnostic criteria for CAP in adults. CAP was defined as the presence of a new radiologic pulmonary infiltrate and the onset of symptoms with at least one of the following indicators: cough, sputum production, and dyspnoea; body temperature > 38.0℃; rales on auscultation; and a peripheral white blood cell count (WBC) > 10 × 10^9^/L or < 4 × 10^9^/L^17^. (3) The patient had positive results for MP. A definitive diagnosis of MPP is defined as a serological MP-immunoglobulin M (IgM) titre ≥ 1:160 or a fourfold increase in antibody titres during the acute and convalescent phases^18^. (4) The patient had complete clinical data. The exclusion criteria were as follows: (1) age < 18 years old; (2) infection with other pathogens; (3) pulmonary tuberculosis, bronchiectasis, bronchial asthma, chronic obstructive pulmonary disease and other pulmonary diseases; (4) severe community-acquired pneumonia; and (5) incomplete clinical data.

Estimates of the sample size were based on the principle of at least 10 outcome events per variable^19,20^, and our sample size was sufficient to yield valid results.

### Data collection

Demographic, clinical, laboratory, and radiographic characteristics were collected within 24 h of admission. The demographic data included sex, age, and season of onset. The clinical data included fever, duration of fever (time from fever onset to hospitalization), body temperature, cough and sputum, dry cough, pharyngeal malaise, shortness of breath, aversion to cold, chills, fatigue, dizziness, headache, muscle soreness, and duration of hospital stay. The laboratory data included white blood cell count (WBC), absolute neutrophil count, lymphocyte count, platelet count (PLT), C-reactive protein (CRP), procalcitonin (PCT), erythrocyte sedimentation rate (ESR), and D-dimer. The radiographic characteristics were analysed independently by two experienced radiologists.

### Ethics statement

This study was approved by the Ethics Committee of Shanxi Bethune Hospital. Written informed consent was obtained from a parent and/or legal guardian of each participant. This study was performed in accordance with the Declaration of Helsinki.

### Statistical analysis

SPSS software version 22.0 (SPSS Inc.) was used to analyse the data. The median and interquartile range (IQR) were used for the quantitative data with a nonnormal distribution. The Mann‒Whitney *U* test was used to compare the two groups. Percentage (%) and cases (n) were used for the enumeration data. The chi-square test (*χ*^2^ test) was adopted for comparisons between the two groups. Binary logistic regression analysis was used to perform a multivariate analysis to obtain independent predictors of MPP in adults. The nomogram prediction model was constructed using the screened independent predictors. The nomogram was constructed and drawn using R software version 4.1.2 (https://www.r-project.org/). Multicollinearity was checked before determining the final model. The nomogram is based on the regression coefficients in the multiple logistic regression, with values ranging from 0 to 100 points, and is a visualization of the regression equation. Each parameter in the nomogram has a corresponding score, and the scores for each parameter are added to obtain the total score. Each total score corresponds to the probability of a clinical event occurring in a given patient^21,22^.

The discriminative ability and prediction accuracy of the nomogram were evaluated by the concordance index (C-index). A C-index is typically between 0.5, 0.5–0.7, 0.7–0.9 and > 0.9, which represents low, medium, high, and very high accuracy. The calibration curve was used to evaluate the actual and predicted risk of the MPP nomogram. The predictive power of the nomogram was assessed by the receiver operating characteristic (ROC) curve, and the area under the ROC curve (AUROC) was calculated. The clinical net benefit was assessed by the decision curve analysis (DCA) curve. Finally, bootstraps with 1000 resamples were used for internal validation. *P* < 0.05 was considered statistically significant.

## Results

### Patient characteristics

In this study, a total of 337 patients met the inclusion and exclusion criteria, including 112 patients in the MPP group and 225 patients in the non-MPP group. The patients were randomly divided into a training set (n = 236) and a validation set (n = 101) at a ratio of 7:3^23^. There were 75 MPP patients and 161 non-MPP patients in the training set. There were 37 MPP patients and 64 non-MPP patients in the validation set.

### Univariate analysis

The results of the univariate analysis of the two groups of patients are shown in Table 1. In the MPP group, the incidence was higher in the females than in the males, and the median age of patients was 33 years. In the non-MPP group, the incidence was higher in the males than in the females, and the median age of patients was 50 years. MPP was more likely to be present in the autumn and winter. There were statistically significant differences in sex, age, and season of onset between the two groups of patients (*P* < 0.05).

**Table 1:** Univariate analysis of MPP in adults.

Variables	MPP	Non-MPP	*P* value
	(n = 112)	(n = 225)	
**Sex**			
Male, n (%)	46 (41.1)	119(52.9)	0.049
Female, n (%)	66 (58.9)	106 (47.1)	
Age(years), median (IQR)	33 (26–37.5)	50(35–64)	< 0.001
**Season of onset, n (%)**			
Spring	6 (5.4)	27 (12)	< 0.001
Summer	15 (13.4)	67 (29.8)	
Autumn	57 (50.9)	86 (38.2)	
Winter	34 (30.4)	45 (20)	
**Clinical manifestations**			
Fever, n (%)	102 (91.1)	150 (66.7)	< 0.001
Duration of fever(d), median (IQR)	4.5 (3–7.5)	3 (1–5)	< 0.001
Body temperature (℃), median (IQR)	39 (38.2–39.5)	38.2(36.5–39)	< 0.001
Cough and sputum, n (%)	74 (66.1)	142 (63.1)	0.631
Dry cough, n (%)	26 (23.2)	19(8.4)	< 0.001
Pharyngeal malaise, n (%)	26 (23.2)	27 (12)	0.011
Shortness of breath, n (%)	13 (11.6)	52 (23.1)	0.013
Aversion to cold, n (%)	57 (50.9)	70 (31.1)	0.001
Chills, n (%)	36 (32.1)	44 (19.6)	0.014
Fatigue, n (%)	46 (41.1)	64 (28.4)	0.026
Dizziness, n (%)	13 (11.6)	9 (4)	0.010
Headache, n (%)	11 (9.8)	32 (14.2)	0.300
Muscle soreness, n (%)	22 (19.6)	28 (12.4)	0.103
Duration of hospital stay (d), median (IQR)	6 (5–8)	7 (6–10)	0.001
**Laboratory data**			
WBC(× 10^9^/L), median (IQR)	6.5 (4.8–8.1)	6.5 (4.9–9)	0.264
Absolute neutrophil (× 10^9^/L), median (IQR)	4.4 (3.1–5.5)	4.4 (3–7)	0.200
Lymphocyte count(× 10^9^/L), median (IQR)	1.3 (0.9–1.7)	1.3 (0.9–1.6)	0.357
PLT(× 10^9^/L), median (IQR)	236.5 (192.5–301)	226 (179–277)	0.069
CRP (mg/L), median (IQR)	28.2 (10.4–55.6)	51.2 (28.6–63)	< 0.001
PCT (ng/ml), median (IQR)	0.07 (0.01–0.15)	0.08 (0.03–0.15)	0.656
ESR (mm/h), median (IQR)	36(20–51)	38 (20–56)	0.412
D-dimer(ng/mL), median (IQR)	416 (201–872)	528 (287–861)	0.035
**Radiographic characteristics**			
Left lung, n (%)	73 (65.2)	157 (69.8)	0.456
Right lung, n (%)	78 (69.6)	172 (76.4)	0.188
Bilateral lungs, n (%)	48 (42.9)	105 (46.7)	0.562
Ground-glass opacities, n (%)	43 (38.4)	100 (44.4)	0.290
Patchy shadows, n (%)	66 (58.9)	139 (61.8)	0.637
Lung consolidations, n (%)	57 (50.9)	93 (41.3)	0.104
Nodular shadows, n (%)	21 (18.8)	45 (20)	0.884
Tree-in-bud sign, n (%)	22 (19.6)	24 (10.7)	0.029
Bronchial wall thickening, n (%)	13 (11.6)	18 (8)	0.318
Air bronchial sign, n (%)	15 (13.4)	19 (8.4)	0.180
Pleural effusions, n (%)	5 (4.5)	30 (13.3)	0.013

*WBC* white blood cell count, *PLT* platelet count, *CRP* C-reactive protein, *PCT* procalcitonin, *ESR* erythrocyte sedimentation rate.

In terms of the clinical manifestations, there were statistically significant differences in fever, duration of fever, body temperature, dry cough, pharyngeal malaise, shortness of breath, aversion to cold, chills, fatigue, dizziness, and duration of hospital stay between the two groups of patients (*P* < 0.05). In the laboratory results, the levels of CRP and D-dimer in the MPP group were lower than those in the non-MPP group, both of which were significantly different (*P* < 0.05). According to imaging examinations, the MPP group had a higher proportion of patients with tree-in-bud signs and a lower proportion of patients with pleural effusion than the non-MPP group, all of which were significantly different (*P* < 0.05).

### Multivariable regression analysis

The significant variables in the univariate analysis were included in the logistic regression model for the multivariate analysis. The multivariate regression analysis showed that age (OR 0.950, 95% CI 0.932–0.969, *P* < 0.001), body temperature (OR 1.658, 95% CI 1.250–2.199, *P* < 0.001), dry cough (OR 2.420, 95% CI 1.144–5.117, *P* = 0.021), dizziness (OR 0.254, 95% CI 1.101–16.971, *P* = 0.012), CRP (OR 0.987, 95% CI 0.979–0.995, *P* = 0.001) and tree-in-bud sign (OR 2.161, 95% CI 1.004–4.654, *P* = 0.049) were independent predictors of MPP in adults. The results are shown in Table 2.

**Table 2:** Multivariable logistic regression analysis of MPP in adults.

Variables	*β*	*OR*	95% CI	*P* value
Age(years)	− 0.051	0.950	0.932–0.969	< 0.001
Body temperature (℃)	0.506	1.658	1.250–2.199	< 0.001
Dry cough	0.884	2.420	1.144–5.117	0.021
Dizziness	1.372	0.254	1.101–16.971	0.012
CRP	− 0.013	0.987	0.979–0.995	0.001
Tree-in-bud sign	0.771	2.161	1.004–4.654	0.049

### The establishment and validation of the nomogram

**Figure 1:**
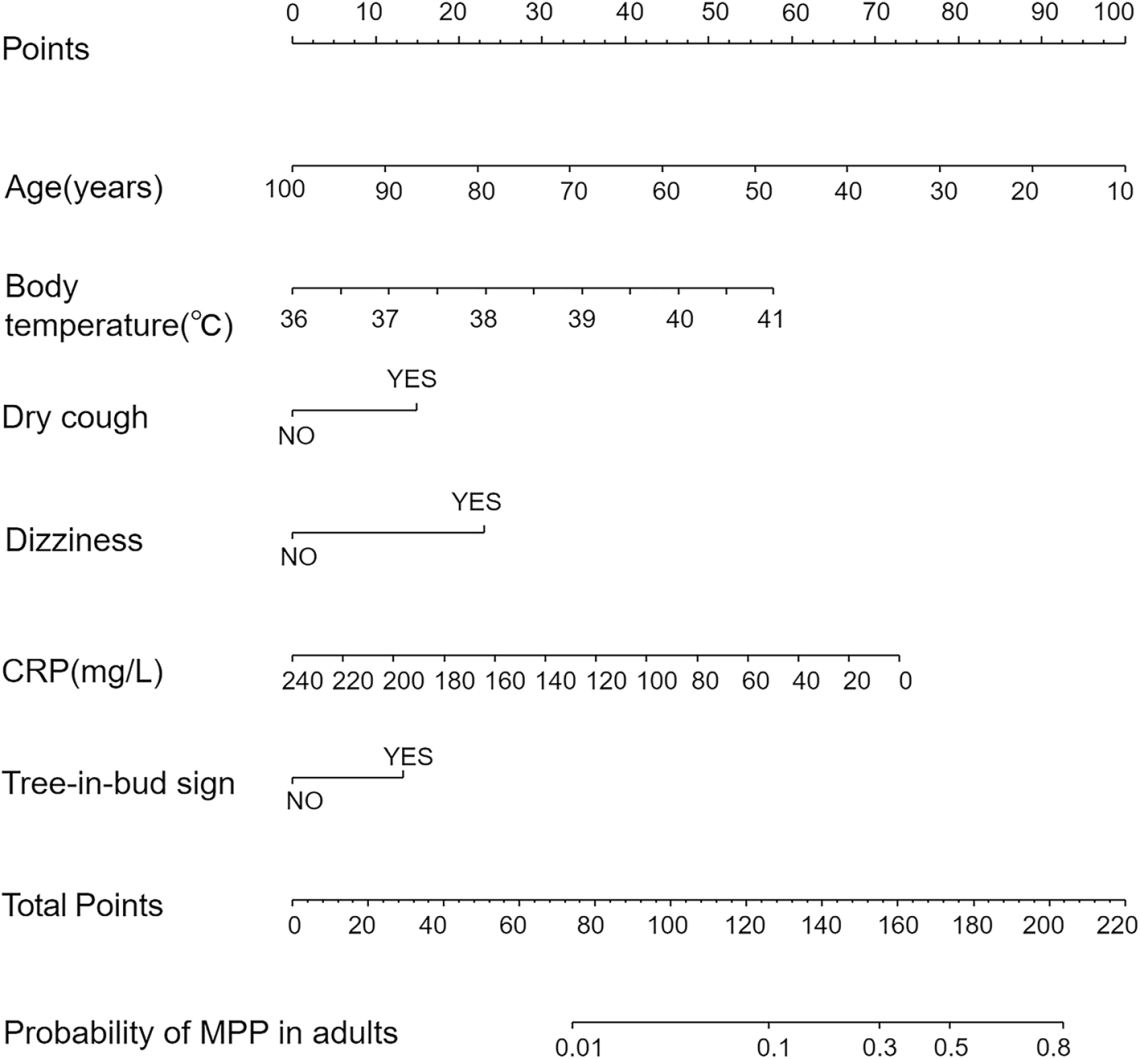
Nomogram to evaluate the probability of MPP in adults. Each variable corresponds to the corresponding point on the corresponding variable axis of the nomogram, and the vertical line of the variable axis is drawn through this point. The corresponding points are obtained on the points axis at the top, and the total points are obtained by adding up the points of all the variables. The point on the probability axis corresponding to the total points is the predictive value of having the disease.

According to the results of the multivariate analysis, we constructed a nomogram model including six predictors: age, temperature, dry cough, dizziness, CRP, and tree-in-bud sign (Fig. 1).

**Figure 2:**
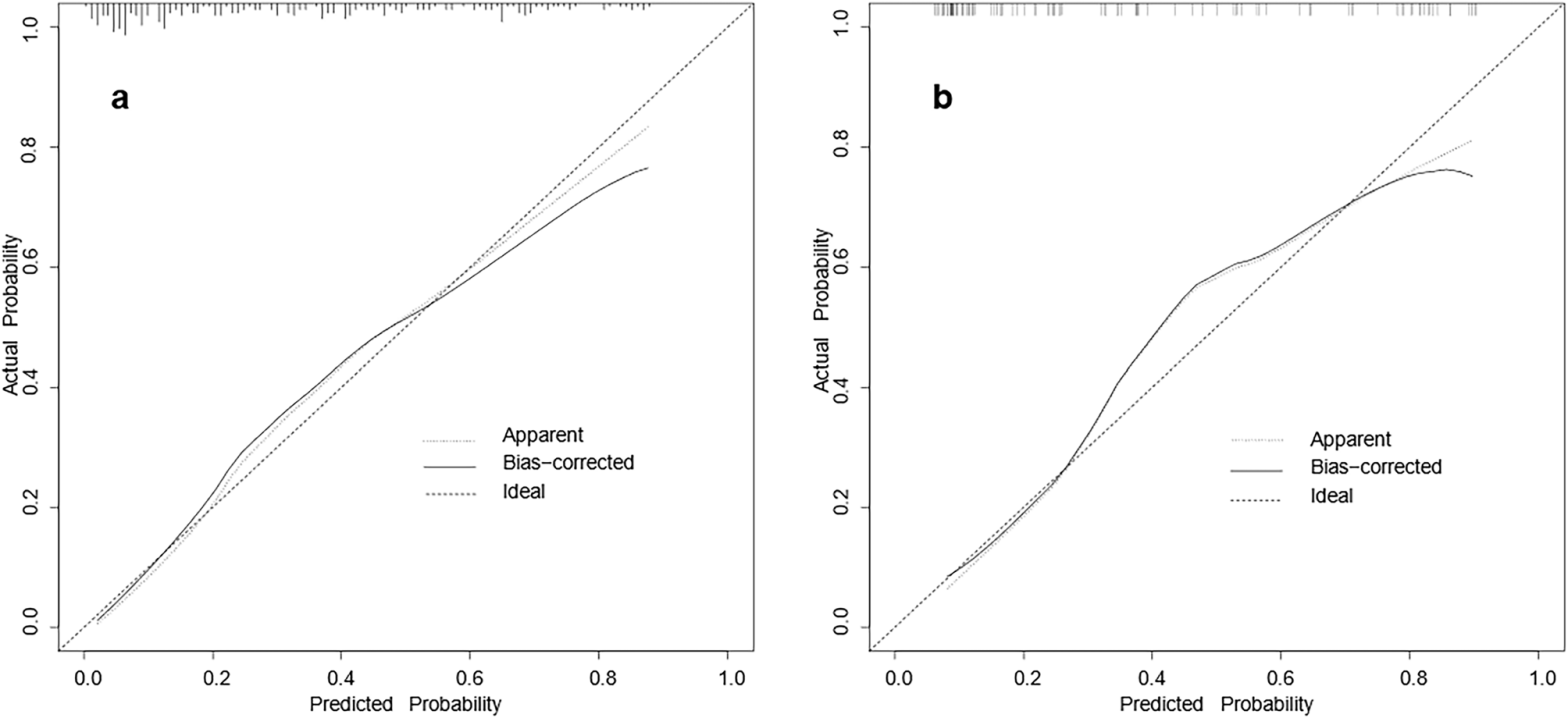
Calibration curves for the nomogram model of MPP in adults ((**a**) training set, (**b**) validation set). The x-axis represents the probability of MPP in adults predicted by the nomogram; the y-axis represents the actual outcome. The diagonal dashed line represents the ideal prediction using the ideal model; the solid black line represents the nomogram model calibration curve. The calibration curve close to the diagonal dashed line indicates good agreement between the predicted and actual probabilities.

**Figure 3:**
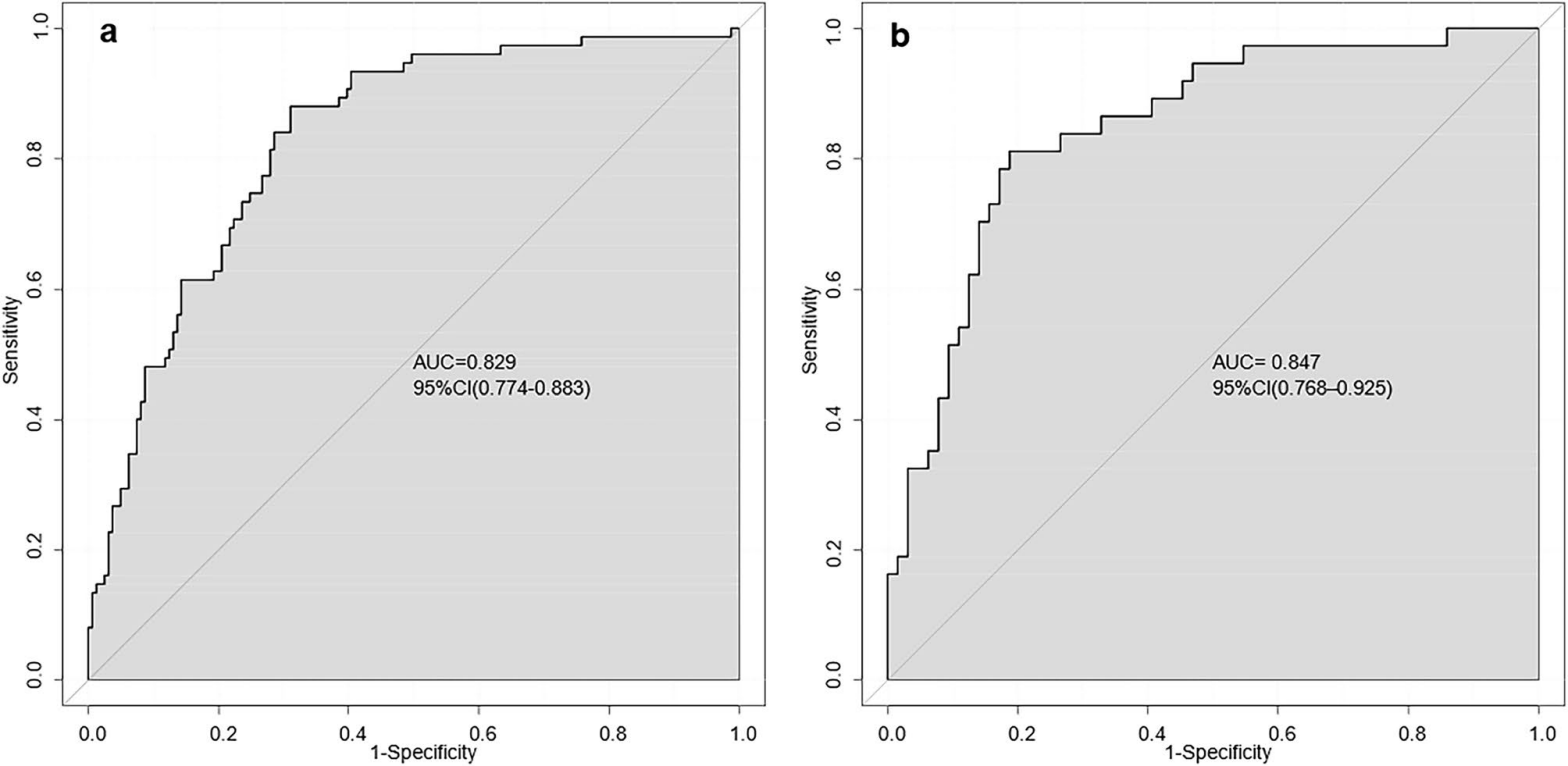
AUROC of the nomogram model ((**a**) training set, (**b**) validation set). The closer the AUROC is to 1, the more reliable the predictive ability of the model.

The final model was validated internally by using the bootstrap method (1000 repetitions). The model had good precision and discrimination with a concordance index (C-index) of 0.837. As shown by the calibration curves, the calibration curves of the nomogram were highly consistent with the standard curves in the training and validation sets (Fig. 2). The AUROC was 0.829 (95% CI 0.774–0.883) in the training set and 0.847 (95% CI 0.768–0.925) in the validation set (Fig. 3), indicating the high reliability of the nomogram’s prediction ability.

### Clinical utility of the nomogram was evaluated by DCA curves

**Figure 4:**
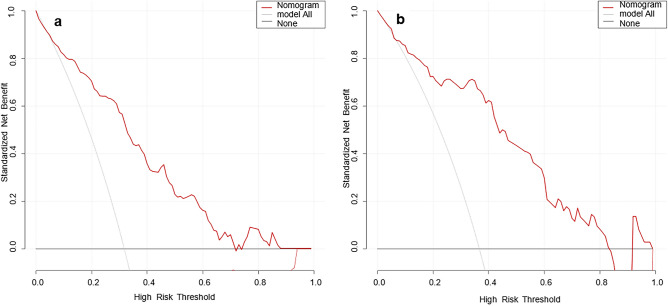
DCA curves of the nomogram model ((**a**) training set, (**b**) validation set). The x-axis is the high-risk threshold probability, and the y-axis is the net benefit. The grey and black lines are the extreme curves; the grey line stands for the hypothesis that all patients suffer from MPP; and the black line stands for the hypothesis that all patients do not suffer from MPP. The red line is the DCA curve of the nomogram model, representing the net benefit of the model under different high-risk thresholds. The farther the DCA curve is from the extreme curve, the greater the range of net benefit and the better the clinical utility.

The DCA curves of the nomogram were shown in Fig. 4. The net benefit of using the nomogram to predict MPP in adults was high when the threshold probability was between 0.02 and 0.71 in the training set (Fig. 4a) or between 0.01 and 0.83 in the validation set (Fig. 4b). Therefore, the nomogram had good clinical utility for predicting MPP in adults.

## Discussion

MPP is a seasonal epidemic. A delayed diagnosis will increase the risk of infection in the surrounding people, and patients with severe MP infection may even need to be admitted to the intensive care unit (ICU), which affects the quality of life of patients. Therefore, it is critical to develop models for the early prediction of MPP in adults.

In this study, which was a case‒control study cohort of 337 patients, a nomogram model was developed to predict the risk of developing MPP in adults. The results showed that age, body temperature, dry cough, dizziness, CRP and tree-in-bud sign were independent predictors of MPP in adults. The nomogram based on these 6 factors showed good predictive performance.

Age was one of the independent predictors of MPP in adults in our study. In a nomogram study of RMPP in children, age was included, and the incidence of RMPP was positively associated with age^24^. However, there was no such association in adult MPP. MPP in adults is more likely to occur in young adults. The median age of the patients in this study was 33 years, similar to previous reports^25,26^. This may be related to the fact that young people have many social activities, and workplaces are mostly indoors with poor air circulation and are associated with people in close contact. At the same time, we found that women are more prone to MPP than men, and the specific reasons need to be further studied.

Fever, dry cough and dizziness are common clinical symptoms of MPP. Patients with MPP usually have different degrees of fever, and most of them are moderate to high^27^. We found that the average body temperature of the patients was 39 °C, which was consistent with previous studies^25^. Insufficient blood supply to the brain during fever in MPP patients may lead to dizziness. Dry cough is secondary to tracheobronchitis caused by the invasion of MP into the respiratory epithelium^28^. A study has reported that facial oedema, chest pain, chest tightness, and dizziness occur when patients with MPP cough violently^29^. In addition, dizziness in MPP patients may also be related to autoimmunity or the formation of immune complexes^8,30^. In our study, the combination of these clinical symptoms in predictive models offers a great potential advantage for predicting the occurrence of MPP in adults.

CRP is an acute-phase reactant that begins to be secreted 4–10 h after an inflammatory injury, peaks at 48 h, and has a half-life of 19 h. The magnitude of its increase was positively correlated with the inflammation severity. Dynamic monitoring of CRP levels can be used to assess the prognosis of hospitalized CAP patients^31^. In a prior investigation, CRP was found to be an independent predictor of refractory mycoplasma pneumonia in children^32^. We found that CRP was a predictor of MPP in adults and was mildly elevated in MPP adult patients, which suggested a mild inflammatory response in patients.

In this study, tree-in-bud signs and bronchial wall thickening were more common in the adult MPP group than in the non-MPP group, which is consistent with previous reports^26,33^. MP adheres to the respiratory mucosal epithelium via an adhesion protein, and it releases toxins that directly damage the respiratory epithelium and cause thickening of the bronchial walls. When lesions are distributed around the bronchioles, mucus and other inflammatory substances block the terminal bronchioles and alveolar sacs, resulting in the tree-in-bud sign^34,35^.

A nomogram is a visual representation of a statistical model that allows for personalized prediction of the incidence of clinical events. In previous studies, nomograms have been used to predict MPP in children^36,37^. However, there are few studies on nomograms of MPP in adults. To our knowledge, this is the first nomogram developed and validated that can be used to predict the risk of incidence of MPP in adults. The six predictors included in this nomogram were derived from routinely collected clinical data, which are available within hours of admission, and this can help clinicians to more quickly identify adults at high risk for MPP.

However, there were some limitations. First, this single-centre retrospective study suffers from an inherent selection bias. Second, we only applied internal validation to evaluate the model, and large-scale, multicentre, prospective studies are needed for external validation before it can be applied to clinical practice. Third, this study is based on the cohort findings of the Chinese population and may not be applicable to patients of other ethnicities. We encourage validation of this model in centres across ethnicities as well as in other regions.

## Conclusion

In conclusion, age, body temperature, dry cough, dizziness, CRP, and tree-in-bud sign were independent predictors of MPP in adults. We constructed a nomogram with a reliable predictive power. The nomogram may be a powerful tool to assist clinicians in making personalized decisions.

## Data Availability

The datasets used during the current study available from the corresponding author on reasonable request.
